# Occurrence of twin embryos in the eastern bluebird

**DOI:** 10.7717/peerj.273

**Published:** 2014-03-11

**Authors:** Robyn L. Bailey, Gerald E. Clark

**Affiliations:** 1 Cornell Lab of Ornithology, Ithaca, NY, USA; 2 Retired, State College, PA, USA

**Keywords:** Pennsylvania, Eastern bluebird, *Sialia sialis*, Twinning, Double-yolked egg, Citizen science

## Abstract

We report the first record of presumed twinning in eastern bluebird (*Sialia sialis*) and provide a review of previously reported twinning events in wild birds. A nest containing twin eastern bluebird nestlings was monitored in 2013 in central Pennsylvania and reported to the Cornell Lab of Ornithology’s NestWatch program, a national program where volunteers submit data on wild nesting birds. A presumed double-yolked egg of a free-living eastern bluebird pair hatched successfully, and twin nestlings lived for 11 days in a nest box shared by three siblings. Due to the rarity of twinning in wild birds, engaging the public to monitor large numbers of nests is the most likely approach to documenting twinning in wild populations, and citizen science provides the infrastructure for individuals to share observations.

## Introduction

 Twin avian embryos occur rarely (Romanoff & Romanoff, 1972; O’Connor, 1984; Pourlis, 2011), yet twinning has been documented in commercial species of poultry, waterfowl and game birds (see Romanoff & Romanoff, 1972, for review). Estimates of double-yolked eggs in poultry range from 0.87% in domestic turkeys (Nestor & Bacon, 1972) to 2.8% in domestic chickens (Jeffrey, Fox & Smyth, 1953). Few studies, however, have documented the rates at which twinning occurs within wild populations, likely because estimating rare events requires large sample sizes. Twinning in free-living birds has been reported for a small but diverse group of species (Bassett *et al.*, 1999). As summarized in Table 1, twinning in wild birds is discovered either by (1) the dissection of unhatched eggs, or (2) by the presence of more nestlings than eggs. Given the rarity of twinning in birds, we document the successful hatching of twin eastern bluebirds (*Sialia sialis*) in a wild population in central Pennsylvania.

**Table 1:** We found 14 documented cases of twinning for 13 species of free-living birds. Examples are known from either dissected unhatched eggs (*n* = 10) or from unassisted, hatched eggs where nestlings exceeded clutch size (*n* = 4).

Species	Source	Hatched? (Y/N)
Adélie penguin, *Pygoscelis adeliae*	Astheimer & Grau, 1985	No
American goldfinch, *Spinus tristis*	Berger, 1953, case 1	Yes
American goldfinch, *Spinus tristis*	Berger, 1953, case 2	Yes
Brown thrasher, *Toxostoma rufum*	Cartwright, 1939	No
Eastern bluebird, *Sialia sialis*	Bailey & Clark, herein	Yes
Gadwall, *Anas strepera*	Lokemoen & Sharp, 1981	No
Giant Canada goose, *Branta canadensis maxima*	Batt, Cooper & Cornwell, 1975	No
Hihi, *Notiomystis cincta*	Thorogood & Ewen, 2006	No
House sparrow, *Passer domesticus*	Griffith & Stewart, 1998	No
Northern goshawk, *Accipiter gentilis*	Petty & Anderson, 1989	No
North Island kaka, *Nestor meridionalis*	Alley & Berry, 2002	No
Peregrine falcon, *Falco peregrinus*	Pattee, Mattox & Seegar, 1984	No
Song sparrow, *Melospiza melodia*	Berger, 1953	Yes
Wedge-tailed shearwater, *Puffinus pacificus*	Pettit & Causey Whittow, 1981	No

According to Romanoff & Romanoff (1972), complete twin embryos may arise from one of at least three recognized ways: (1) monovular twins, usually from a normal-sized egg, containing one blastoderm on one yolk (monozygotic identical twins of the same sex resulting either from double gastrulation or from longitudinal fission), (2) monovular twins with two blastoderms on one yolk (bizygotic fraternal twins of either sex), or (3) binovular twins from a large double-yolked egg (fraternal twins resulting from two yolks meeting in the oviduct and becoming enclosed in one egg). Crowding in a double embryo egg is likely a significant barrier to hatching, since at least one chick must be able to bring the beak in contact with the shell for pipping (Hollander & Levi, 1940). Hatching may also prove fatal if the embryos are not positioned with access to the air cell or if the yolk sac becomes ruptured during hatching (Romanoff & Romanoff, 1972; Bassett *et al.*, 1999).

Jeffrey, Fox & Smyth (1953) reported that two of 152 fertile double-yolked chicken eggs (1.3%) survived long enough to pip the egg, but only one was able to hatch with assistance. Hollander & Levi (1940) found that about 14% of double-yolked pigeon eggs survived to hatching stage, but none were able to hatch successfully. In one extraordinary account, Bernard (1850) claimed that nine of 10 double-yolked eggs from the same chicken hatched 18 chicks when incubated (as cited in Jeffrey, Fox & Smyth, 1953), but this high hatchability has never been replicated. Therefore, it is likely that very few double embryo eggs can actually hatch without assistance.

Our observation appears to be an extremely rare case, in which an egg containing two viable twins survived the incubation period, hatched without assistance, and survived for at least 11 days. This observation was made by a volunteer participant (GC) in NestWatch, a citizen-science project administered (by RB) at the Cornell Lab of Ornithology (www.nestwatch.org). Citizen science may provide future opportunities to study this rare phenomenon, since a number of nest record schemes already exist around the world. Due to the rarity of twinning in wild birds, engaging the public to monitor large numbers of nests is the most likely approach to documenting twinning in wild populations.

## Methods

A NestWatch participant (GC) routinely monitored an eastern bluebird nest from 17 June through 21 July 2013, using a mobile phone equipped with a camera to capture images of the nest throughout the nesting period. The nest was located in a nest box designed to attract eastern bluebirds. The nest box was made of 1.6-cm-thick pine lumber and had internal dimensions of 10.5 × 9.5 × 24.8 cm (length × width × height). It was mounted at 1.35 m high and oriented 115° southeast. This nest box was installed at the private residence of GC in State College, Pennsylvania in 2001. The nest was situated in a residential neighborhood of Centre County, where the immediate surrounding vegetation type was human-modified residential: mowed grass with few young trees and cultivated flower beds, and agricultural fields located ∼0.2 km away. The elevation of the site was approximately 356 m. There were two additional nest boxes on the property at the time, both vacant. Supplemental food and water were provided on the premises as live and dried mealworms and a birdbath, which the pair did use.

**Figure 1:**
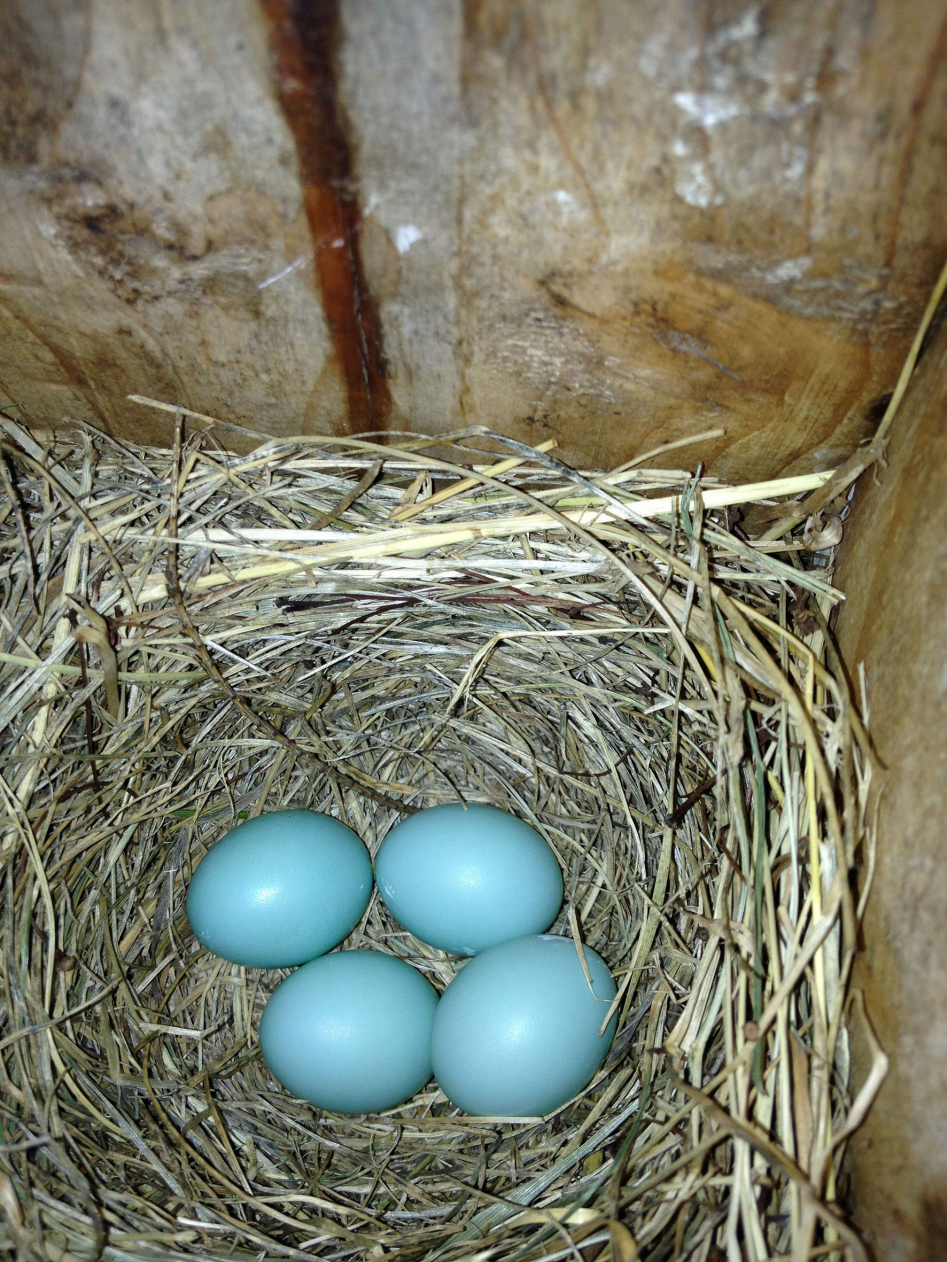
On 17 June 2013, an eastern bluebird (*Sialia sialis*) nest was observed containing one relatively large egg (bottom right) and three normal-sized eggs in State College, Pennsylvania.

Because NestWatch participants typically do not obtain permits to handle nest contents during routine nest monitoring, no physical measurements were made on the eggs or nestlings. Our analysis is therefore based on the interpretation of digital photos of the nest chronology and written notes taken during the active nesting period (Table S1). As one egg was presumed to be a double-yolked egg on the basis of its relatively large size, we measured the length and breadth (in pixels) of all four eggs visible in a photograph (Fig. 1) using the straight line tool and measuring function of ImageJ software (ImageJ version 1.47, http://imagej.nih.gov/ij/, accessed 3 Dec 2013).

We searched the literature to determine if twinning had ever been reported in this species. We used the following keywords to search the Thomson Reuters’ Web of Knowledge database (http://apps.webofknowledge.com) and the Birds of North America Online database (http://bna.birds.cornell.edu): “eastern bluebird”, “avian”, “twin”, “embryo”, “blastoderm”, “double yolk”, and “fertilize”. We also queried the NestWatch citizen-science database at the Cornell Lab of Ornithology (www.nestwatch.org; formerly “The Birdhouse Network”), which contained nest attempt records collected by citizen scientists, to determine if twinning or double-yolked eggs were reported as comments by any of the volunteer nest monitors who have submitted nest observations online since 1997. Research was conducted under the Cornell University Institutional Animal Care and Use Protocol # 2008–0083.

## Results

**Figure 2:**
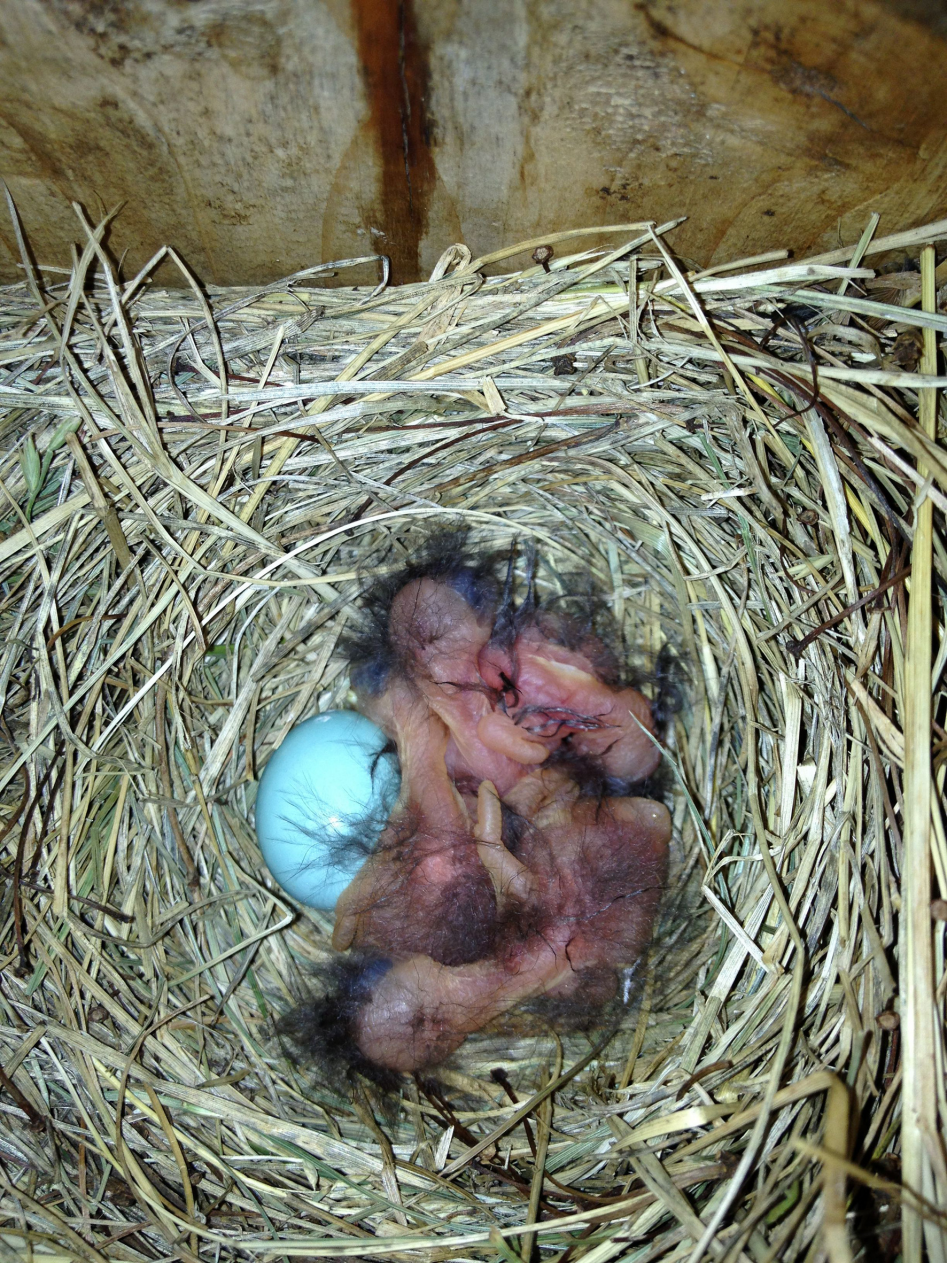
On 1 July 2013, likely the day of hatching, four nestlings were observed with one unhatched egg.

**Figure 3:**
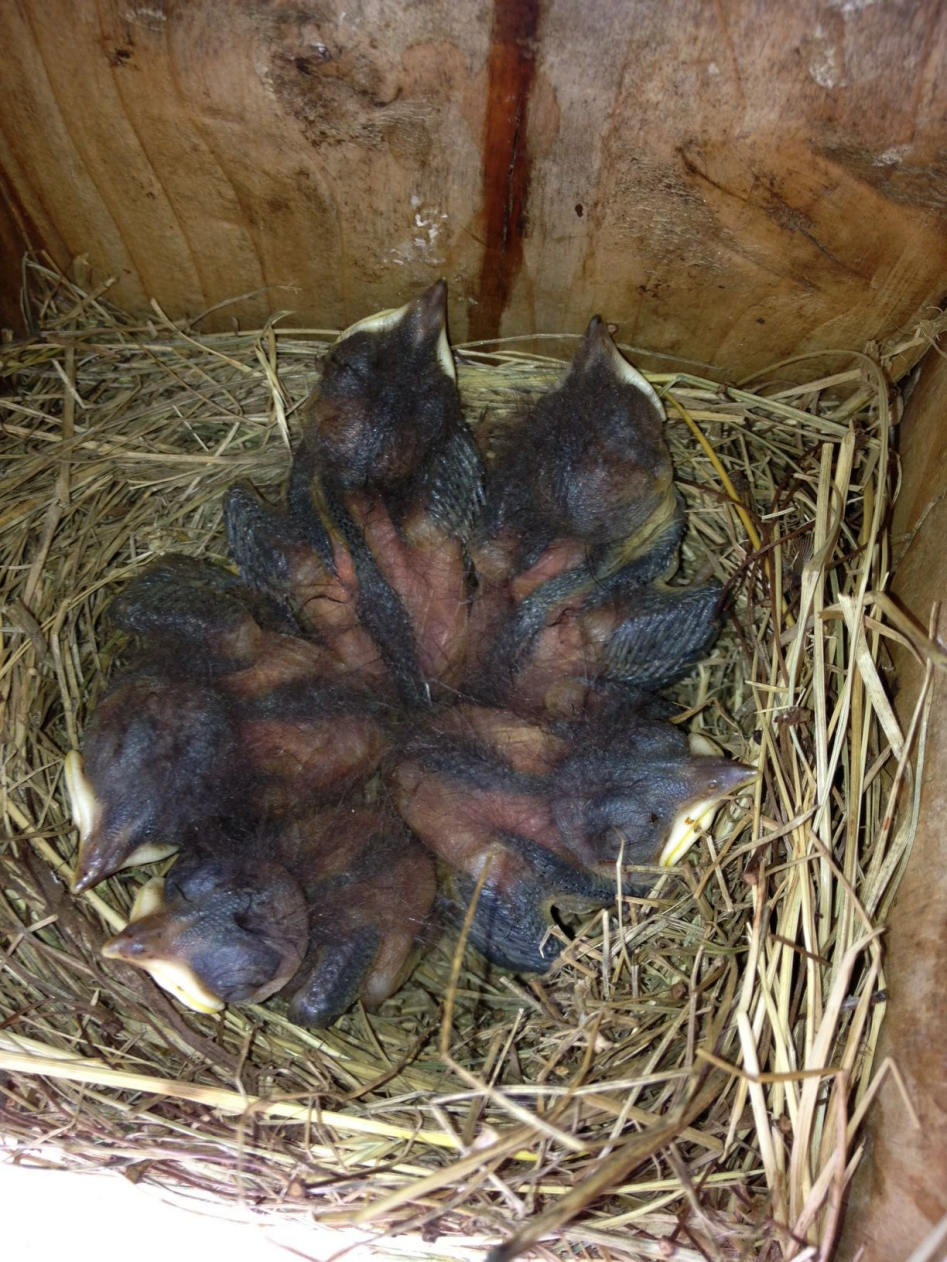
Five eastern bluebird (*Sialia sialis*) nestlings were present on 7 July 2013.

**Figure 4:**
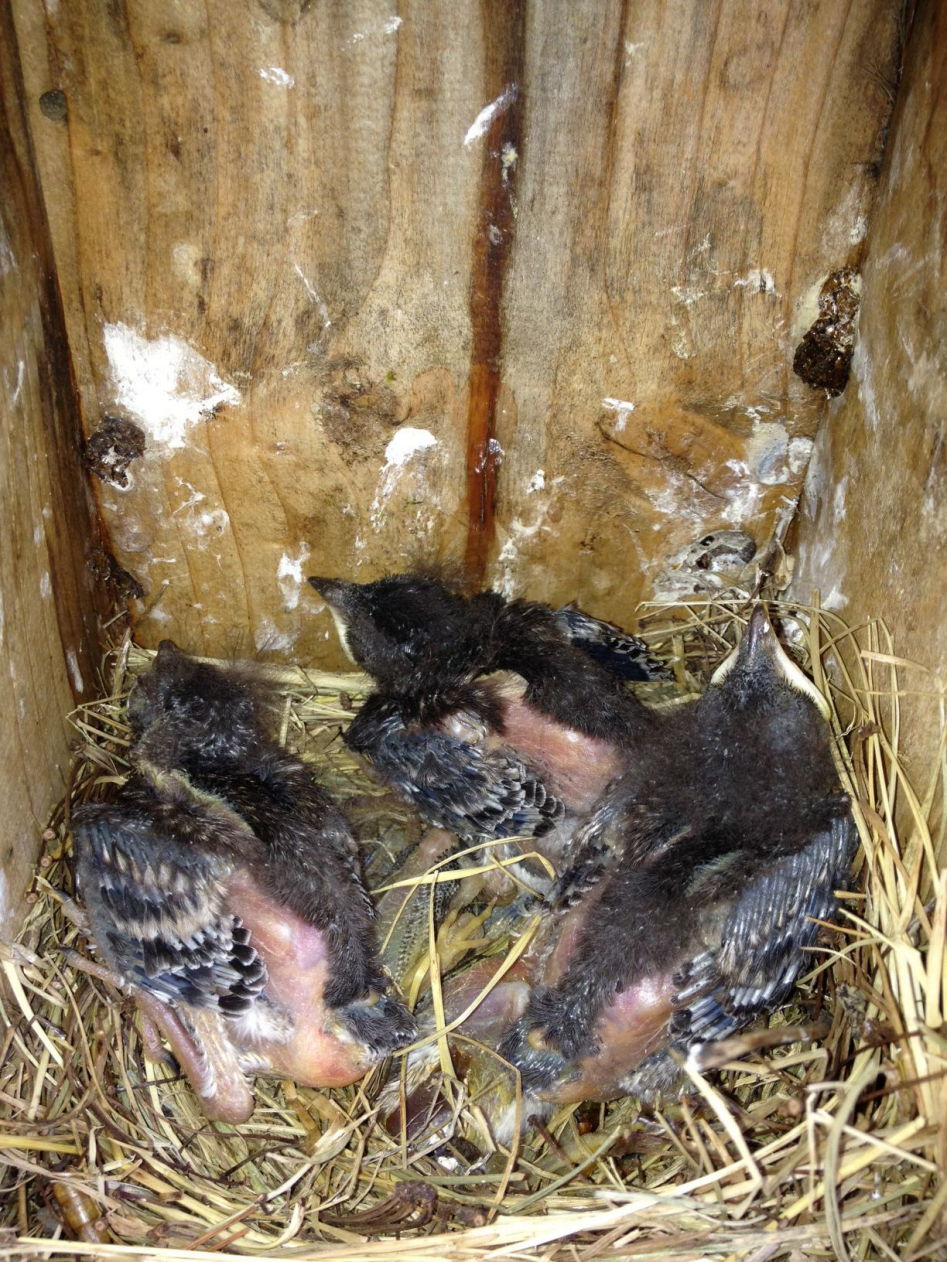
At 3.48 pm on 11 July 2013, the nest contained three live nestlings with two dead nestlings below them.

The nest was discovered on 17 June 2013 with four eggs, one of which was noticeably larger than the others (Fig. 1). Based on an image analysis, the larger egg was approximately 11% longer and 12% wider than the mean of the other three eggs (as measured in pixels), which is within the range reported for double-yolked eggs of other species (Jeffrey, Fox & Smyth, 1953; Pattee, Mattox & Seegar, 1984; Deeming, 2011; Salamon & Kent, 2013). All eggs were the same pale blue color, characteristic of the species. Only four eggs were visible on four subsequent visits to the nest during the egg stage, and eastern bluebirds rotate their eggs during incubation, so it is unlikely that a fifth egg went unnoticed. On 1 July at 5:43 pm, four nestlings were observed likely on the day of hatching, with one egg yet to hatch (Fig. 2). Therefore, we know that the egg containing twin embryos was not the last to hatch. On 7 July, five nestlings were alive and easily counted in the nest, all arranged with their bills facing the outside of the nest, dorsal side up (Fig. 3). By 3.48 pm on July 11, three nestlings were still alive; however two dead nestlings were below them (Fig. 4).

**Figure 5:**
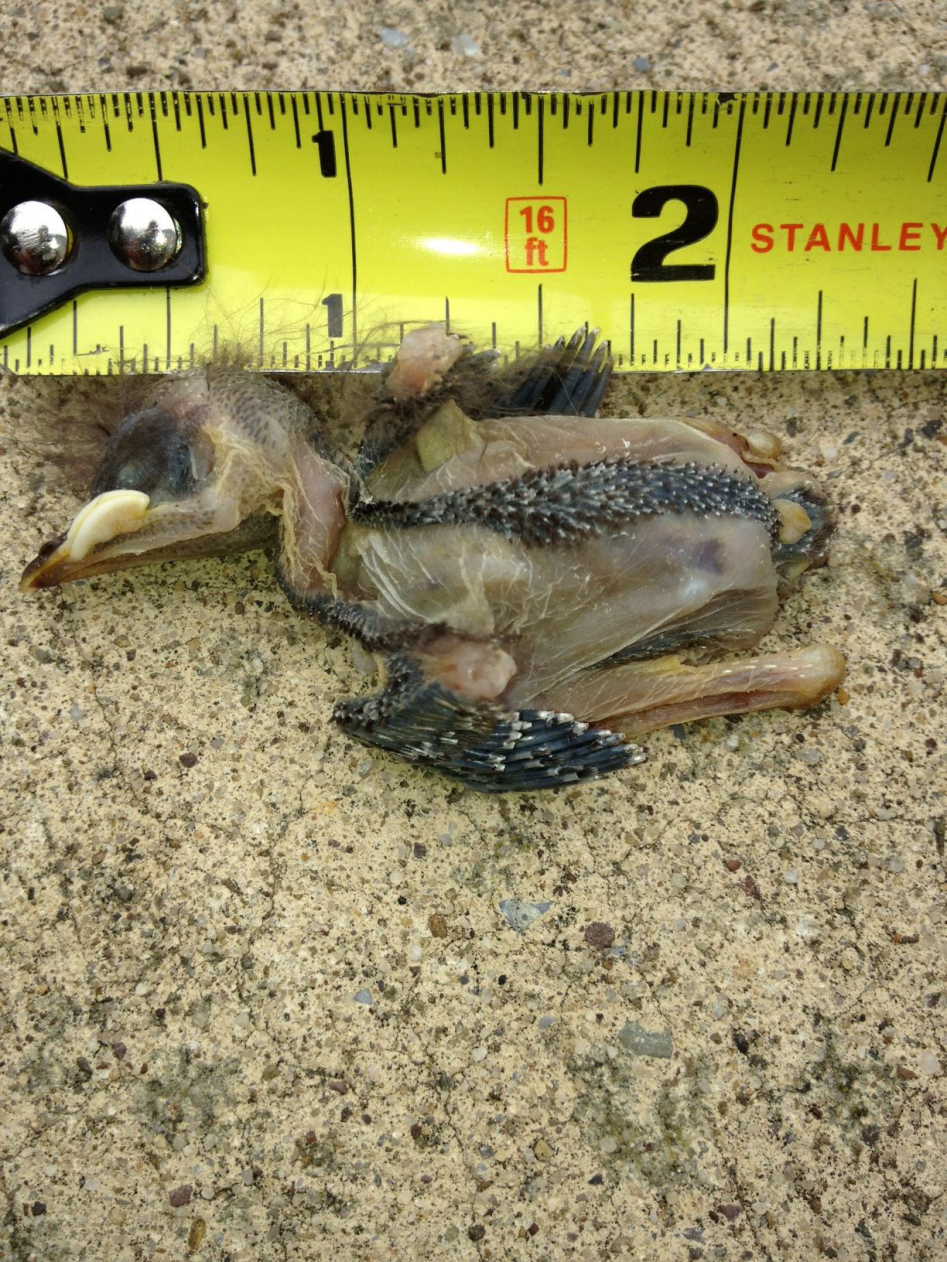
On 12 July 2013, one carcass was found outside the nest box. It showed no obvious signs of structural problems or trauma.

As the one remaining parent, the female removed both carcasses by 10.30 am on 12 July, one of which was found later the same day ∼30.5 m from the nest box. The recovered carcass had no obvious physical injuries or deformities (Fig. 5), and the cause of death is unknown. The male of the pair was not observed after 8 July, raising the possibility that the two nestlings may have starved due to sibling competition for food, despite supplemental feeding (Werschkul & Jackson, 1979). One of the remaining nestlings fledged on 20 July, followed by the other two on 21 July. Assuming the two dead nestlings would have fledged with their siblings, they died 9–10 days prior to fledging.

We found no instances of eastern bluebird twins in our search of the literature, although 12 other species were found (Table 1). Nor did we find any mention of twins or double-yolked eggs for any of the 252 species present in our search of the NestWatch database (*n* = 165, 269 nests as of 21 Nov 2013). Out of 51,790 discrete eastern bluebird nests, only one record mentions a “very large blue egg” in a clutch of four that failed in the egg stage. The large blue egg could have been a double-yolked egg, a large single-yolked egg, or the egg of another species (e.g., European starling, *Sturnus vulgaris*).

## Discussion

To our knowledge, this is the first report of a twin-containing egg of an eastern bluebird, and this expands the number of free-living, unassisted hatched twin cases from three to four. In two cases of twin American goldfinches (*Spinus tristis*), one pair lived for less than four days before the nest was destroyed, but the other case resulted in four fledged young (17 days old) and two dead young (approximately 12 days old), and it is unknown whether the twins were among the fledglings or the deceased (Berger, 1953). Berger (1953) also reported a case of twin song sparrows (*Melospiza melodia*) in which one twin survived to hatching while the other did not; a nestling thought to be the hatched twin (on the basis of its smaller size relative to its nest mate) died at less than four days old. Although it is possible that these bluebird twins were not among the two that were found dead on 11 July, others have reported that twins tend to be smaller than their siblings (Nalbandov, 1942; Bassett *et al.*, 1999).

We speculate that the eastern bluebird twins likely resulted from a double-yolked egg (binovular twins) since the nest contained a relatively large egg, and according to Romanoff & Romanoff (1972), monovular twins are more likely to result from a normal-sized egg (but see Petty & Anderson (1989) and Bassett *et al.* (1999) for exceptions). Double-yolked eggs, which tend to be larger and heavier than single-yolked eggs (Pattee, Mattox & Seegar, 1984; Deeming, 2011; Salamon & Kent, 2013), form when two yolks ovulated within three hours of each other become enclosed in one egg (Conrad & Warren, 1940). According to data on embryonic death in the double-yolked eggs of domesticated fowl, there are two mortality peaks during embryo development, one during days 7–14 of incubation, and another from days 18–23 of incubation; only 3.8% of double-yolked eggs survived to hatching stage (Romanoff & Romanoff, 1972).

Because bluebirds and other cavity-nesting birds are the focus of many conservation projects, especially managed nest box trails, opportunities exist for citizen scientists to contribute to our understanding of rare (or rarely-detected) phenomena, such as twinning in wild birds. Rare events and events that are rarely detected may be more easily studied through the cumulative efforts of dispersed networks of citizen scientists who, collectively, contribute many thousands of hours of work every year (Cooper, Hochachka & Dhondt, 2012). As more people participate in citizen science nest record schemes, like NestWatch, opportunities to study twinning in free-living birds may be forthcoming.

## Supplemental Information

10.7717/peerj.273/supp-1 Supplemental Information 1 Nest visits (extracted from NestWatch.org) documenting the chronology of an Eastern Bluebird nest attempt containing twin embryos.

## References

[ref-1] Alley MR, Berry R (2002). Twinning and embryonic mortality in a free-living kaka (*Nestor meridionalis*). New Zealand Veterinary Journal.

[ref-2] Astheimer LB, Grau CR (1985). Apparent double blastoderms in Adélie penguin eggs. Condor.

[ref-3] Bassett SM, Potter MA, Fordham RA, Johnston EV (1999). Genetically identical avian twins. Journal of Zoology.

[ref-4] Batt BDJ, Cooper JA, Cornwell GW (1975). The occurrence of twin waterfowl embryos. Condor.

[ref-5] Berger AJ (1953). Three cases of twin embryos in passerine birds. Condor.

[ref-6] Bernard C (1850). Discussion on ‘Anomalie d’oeuf de poule’. Comptes rendus des séances de la Société de Biologie.

[ref-7] Cartwright BW (1939). Twin embryos in a brown thrasher egg. Canadian Field Naturalist.

[ref-8] Conrad RM, Warren DC (1940). The production of double yolked eggs in the fowl. Poultry Science.

[ref-9] Cooper CB, Hochachka WM, Dhondt AA, Dickinson JL, Bonney R (2012). The opportunities and challenges of citizen science as a tool for ecological research. Citizen Science: Public participation in environmental research.

[ref-10] Deeming DC (2011). Double-yolked pheasant eggs provide an insight into the control of albumen secretion in bird eggs. British Poultry Science.

[ref-11] Griffith SC, Stewart R (1998). Genetic confirmation of non-identical embryonic twins in the House Sparrow *Passer domesticus*. Journal of Avian Biology.

[ref-12] Hollander WF, Levi WM (1940). Twins and late embryonic monstrosities in pigeons. Auk.

[ref-13] Jeffrey FP, Fox TW, Smyth JR (1953). Observations on double-yolked eggs from the domestic fowl. Journal of Heredity.

[ref-14] Lokemoen JT, Sharp DE (1981). Occurrence of twin gadwall embryos. Condor.

[ref-15] Nalbandov A (1942). A case of viable twin chicks. Journal of Heredity.

[ref-16] Nestor KE, Bacon W (1972). Production of defective eggs by egg and meat type turkey hens. Poultry Science.

[ref-17] O’Connor RJ (1984). The growth and development of birds.

[ref-18] Pattee OH, Mattox WG, Seegar WS (1984). Twin embryos in a peregrine falcon egg. Condor.

[ref-19] Pettit TN, Causey Whittow G (1981). Embryonic double monster in the wedge-tailed shearwater. Condor.

[ref-20] Petty SJ, Anderson DIK (1989). Egg measurements from a northern goshawk (*Accipiter gentilis gentilis*) including one abnormally large egg with twin embryos. Journal of Raptor Research.

[ref-21] Pourlis AF (2011). Developmental malformations in Avian species. Manifestations of unknown or genetic etiology–a review. Asian Journal of Animal and Veterinary Advances.

[ref-22] Romanoff AL, Romanoff AJ (1972). Pathogenesis of the avian embryo.

[ref-23] Salamon A, Kent JP (2013). Double and single yolked duck eggs: their contents and dimensions compared and the mechanical stimulation hypothesis for albumen secretion is supported. International Journal of Poultry Science.

[ref-24] Thorogood R, Ewen JG (2006). Rare occurrence of embryonic twins in the Hihi (Stitchbird) *Notiomystis cincta*: an endangered passerine of New Zealand. Ibis.

[ref-25] Werschkul DF, Jackson JA (1979). Sibling competition and avian growth rates. Ibis.

